# Chemotherapy with cisplatin: insights into intracellular pH and metabolic landscape of cancer cells in vitro and in vivo

**DOI:** 10.1038/s41598-017-09426-4

**Published:** 2017-08-21

**Authors:** Marina V. Shirmanova, Irina N. Druzhkova, Maria M. Lukina, Varvara V. Dudenkova, Nadezhda I. Ignatova, Ludmila B. Snopova, Vladislav I. Shcheslavskiy, Vsevolod V. Belousov, Elena V. Zagaynova

**Affiliations:** 1 Institute of Biomedical Technologies, Nizhny Novgorod State Medical Academy, 10/1 Minin and Pozharsky Sq., 603005 Nizhny Novgorod, Russia; 2 Institute of Biology and Biomedicine, Lobachevsky State University of Nizhny Novgorod, 23 Gagarin Ave., 603950 Nizhny Novgorod, Russia; 3 Becker & Hickl GmbH, Nahmitzer Damm 30, 12277 Berlin, Germany; 4 Molecular technologies laboratory, Shemyakin–Ovchinnikov Institute of Bioorganic Chemistry RAS, 16/10 Miklukho-Maklaya St., 117997 Moscow, Russia

## Abstract

Although cisplatin plays a central role in cancer chemotherapy, the mechanisms of cell response to this drug have been unexplored. The present study demonstrates the relationships between the intracellular pH (pHi), cell bioenergetics and the response of cervical cancer to cisplatin. pHi was measured using genetically encoded sensor SypHer2 and metabolic state was accessed by fluorescence intensities and lifetimes of endogenous cofactors NAD(P)H and FAD. Our data support the notion that cisplatin induces acidification of the cytoplasm early after the treatment. We revealed *in vitro* that a capacity of cells to recover and maintain alkaline pHi after the initial acidification is the crucial factor in mediating the cellular decision to survive and proliferate at a vastly reduced rate or to undergo cell death. Additionally, we showed for the first time that pHi acidification occurs after prolonged therapy *in vitro* and *in vivo*, and this, likely, favors metabolic reorganization of cells. A metabolic shift from glycolysis towards oxidative metabolism accompanied the cisplatin-induced inhibition of cancer cell growth *in vitro* and *in vivo*. Overall, these findings contribute to an understanding of the mechanisms underlying the responsiveness of an individual cell and tumor to therapy and are valuable for developing new therapeutic strategies.

## Introduction

Cisplatin [cis-diamminedichloroplatinum(II)] has been in widespread use for many years to treat several forms of cancer, including testicular, ovarian, cervical, head and neck, and non-small-cell lung cancers^1^, however, resistance to this drug remains a major obstacle in chemotherapy.

It is generally accepted that the cytotoxicity of cisplatin is determined primarily by its DNA adducts. DNA damage arrests the cell cycle, inhibits transcription, and initiates cell death (apoptosis or necrosis). However, recent studies suggest that cisplatin has multiple cellular targets beyond nuclear DNA, including membrane lipids^2^ and proteins^3^, mitochondrial proteins and DNA^4^, components of the cytoskeleton^5^, cellular enzymes^6^. Consequently, cell response to cisplatin cannot be fully described in terms of only DNA adduct formation, but can include multiple drug-induced physiological changes. Uncovering the mechanism and targets of cisplatin action can be a key to the understanding of its cytotoxicity and resistance and to the development new therapeutic strategies.

Intracellular pH (pHi) and energy metabolism are thought to contribute to the responses of cancer cells to chemotherapy with cisplatin.

A “reversed” pH gradient with lower (acidic) extracellular pH (pHe) and higher (slightly alkaline or neutral) intracellular pH (pHi) is a common feature of most solid tumors^7^. It has been known for many years that most cancers rely primarily on glycolysis, both aerobic and anaerobic, for energy production^8^. An elevated rate of glycolysis, accompanied by lactate and H^+^ extrusion, leads to extracellular acidosis and alkalization of the cell cytoplasm. In turn, in the presence of adequate levels of oxygen, pHi plays an important role in determining the way cells obtain energy - an alkaline pH driving aerobic glycolysis while an acidic pH drives oxidative phosphorylation (OXPHOS)^9^.

Alkaline pHi has been reported to mediate the resistance of tumor cells to cisplatin^10–12^. One of the possible mechanisms of increased pHi in the cisplatin-resistant lines is overexpression of the vacuolar proton pump V-ATPase. It has been suggested that increased pHi favors the formation of electroneutral species of the drug that have a greater freedom to diffuse within the cell and back out across the cell membrane, resulting in less DNA binding^11^. In spite of numerous data showing that cytoplasmic pH is one of the critical parameters for effective cancer therapy with cisplatin, current knowledge about pHi alterations induced by cisplatin is rudimentary. Rebillard *et al*. has shown that early after cisplatin treatment Na+/H+ membrane exchanger-1 (NHE1) was inhibited in HT29 cells leading to intracellular acidification that promoted apoptosis induction^3^. However, it is unclear whether this observation can be extended to other cancer cell lines and solid tumors.

The link between energy metabolism and cisplatin action is complex. On the one hand, inhibition of glycolysis and a shift toward mitochondrial glucose oxidation upon chemotherapy with cisplatin have been reported for cellular models and tumors^13–17^. An increased oxidative metabolism has been detected in some cisplatin-resistant cell lines^18–21^, suggesting that OXPHOS helps cancer cells to survive. On the other hand, cisplatin is known to affect mitochondrial function^22–24^. Namely, it reduces the activity of the mitochondrial respiratory chain complexes, impairs electron transport chain function, thus inhibiting OXPHOS and adenosine triphosphate (ATP) production, and induces the generation of intracellular reactive oxygen species.

Therefore, the relationships between the pHi, bioenergetics and response of cancer cells to cisplatin have not been fully elucidated. If cellular metabolism and/or pHi are fundamentally altered or affected by cisplatin treatment this may afford a new strategies for combined therapies with the use of metabolic and/or pHi modifying agents.

Modern fluorescence imaging technologies offer versatile opportunities for non-invasive, dynamic, real-time assessments of pHi and the metabolic state of living cells and tissues at a microscopic level.

To measure pHi, genetically encoded sensors based on fluorescent proteins are becoming essential tools. Owing to their stable expression in the cell cytoplasm or in cellular organelles, genetically encoded pH sensors provide the most physiologically relevant information on the spatial and temporal behavior of pH homeostasis^25^. The possibility of long-term monitoring of pHi without the need for loading a dye into the cell gives them a serious advantage over conventional chemical probes. Recently, the new ratiometric (dual excitation) pH-sensor SypHer-2 based on the cpYFP fluorophore has been engineered by Matlashov *et al*.^26^ SypHer2 has a 2 to 3 fold brighter fluorescence signal compared to SypHer and an identical pH sensitivity (pKa 8.1). Using this sensor, we developed a method for pHi mapping in living cancer cells in monolayer cell culture, in 3D spheroids and in tumor xenografts^27^.

Optical metabolic imaging relying on the endogenous fluorescent cofactors, reduced nicotinamide adenine dinucleotide (phosphate) (NAD(P)H) and oxidized flavin adenine dinucleotide (FAD+), is an established approach used to characterize cellular energy metabolism^28, 29^. NADH is produced during glycolysis and the tricarboxylic acid (TCA) cycle via the reduction of NAD+. Molecules of the cofactors NADH and FAD act as electron donors and acceptors, respectively, in the mitochondrial electron transport chain. Two main approaches based on the recording of NAD(P)H and FAD fluorescence are commonly used – (1) the “redox ratio”, which is the ratio of the fluorescence intensities of FAD and NAD(P)H^30^, and (2) measurements of their fluorescence lifetimes^31, 32^. These two methods are complementary to each other as the “redox ratio” reflects the general metabolic rate of the cell while the fluorescence lifetime indicates the state of the cofactor (“free” or “protein-bound”). If no other processes (e.g. biosynthetic pathways or oxidative stress) contribute significantly to the concentration or state of the cofactors, an increased level of glycolysis leads to a decreased redox ratio FAD/NAD(P)H, increased NAD(P)H mean fluorescence lifetimes, and an increased contribution of protein-bound NAD(P)H, and this is typically observed in cancers^13, 33–35^.

The aim of our study was to elucidate the relationships between pHi, energy metabolism and cancer cell response to chemotherapy with cisplatin *in vitro* and *in vivo*.

To analyze pHi, the genetically encoded fluorescent sensor SypHer2 was used. Metabolic activity of the cells was assessed on the basis of the fluorescence intensity and lifetime measurements of the metabolic cofactors NAD(P)H and FAD. *In vitro*, dynamic pHi and metabolic assessments were performed in individual viable cancer cells during exposure to the drug with a focus on the early changes, preceding the manifestation of cytotoxic effects. *In vivo*, pHi and NAD(P)H were measured in tumor xenografts at the end of treatment. The response to chemotherapy was confirmed by cell proliferation assays and live/dead cell staining *in vitro*, and by evaluation of tumor growth and the histopathology of the tumor tissues. To the best of our knowledge, this is the first time that pHi and metabolic changes have been investigated in parallel in individual cancer cells in the course of chemotherapy and correlated with the cell and tumor responses.

## Results

### Cell viability and proliferation under cisplatin exposure

**Figure 1:**
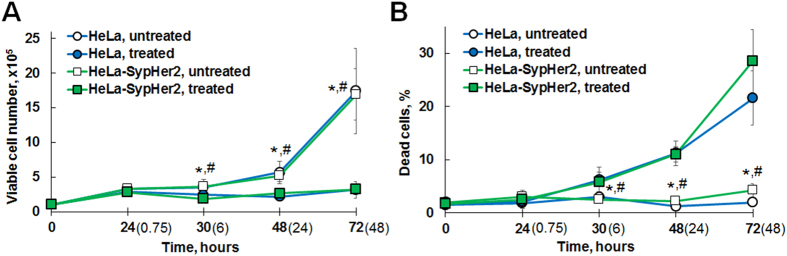
Effect of cisplatin on the proliferative activity and survival of cancer cells. (**A**) Viable cell number for HeLa and HeLa-SypHer2 cells. (**B**) Percentage of dead (trypan blue positive) cells. The cells (1 × 10^5^) were seeded at “0 hours” time-point and cisplatin was added 24 hours after cell seeding. The cells were exposed to cisplatin for 45 min, 6, 24 or 48 hours, the duration of cisplatin exposure is indicated in parentheses. Untreated cells served as control. Mean ± SD. All assays were repeated in triplicate in two independent experiments. *Statistically significant difference between treated and untreated HeLa cells, *p* ≤ 0.05. ^#^Statistically significant difference between treated and untreated HeLa-SypHer2 cells, *p* ≤ 0.05.

As shown in Fig. 1, treatment with cisplatin inhibited proliferation and increased the percentage of dead cells compared to the untreated controls in a time-dependent manner. At 6-hours exposure, a slight, but statistically significant, decrease in the proliferation rate was observed for both, the HeLa (*p* = 0.042) and HeLa-SypHer2 (*p* = 0.015), cell lines. The percentage of dead cells in the treated plates was from ~3% to ~6% greater than in the untreated controls. On further incubation with cisplatin, the total cell number in the plates did not change, indicating division had been arrested as a result of the treatment, while the number of dead cells increased to 11% after 24 hours, and then to 20–30% after 48-hours of incubation with cisplatin.

Therefore, these data demonstrate that inhibition of HeLa cell growth caused by cisplatin at 2.4–2.6 µM concentration is mediated, to a larger degree, by a reduction in cell proliferation and, to a smaller extent, by a reduction in viability.

### Monitoring pHi in cancer cells *in vitro* under cisplatin exposure

Owing to the stable expression of the pH-sensor in the cell cytoplasm, we could track the changes in pHi values in the individual cancer cells during cisplatin treatment. To explore the relationships between the pHi dynamics and the cellular responses to cisplatin, pHi was analyzed separately in living cells that further showed inhibited proliferation and those that subsequently died.

**Figure 2:**
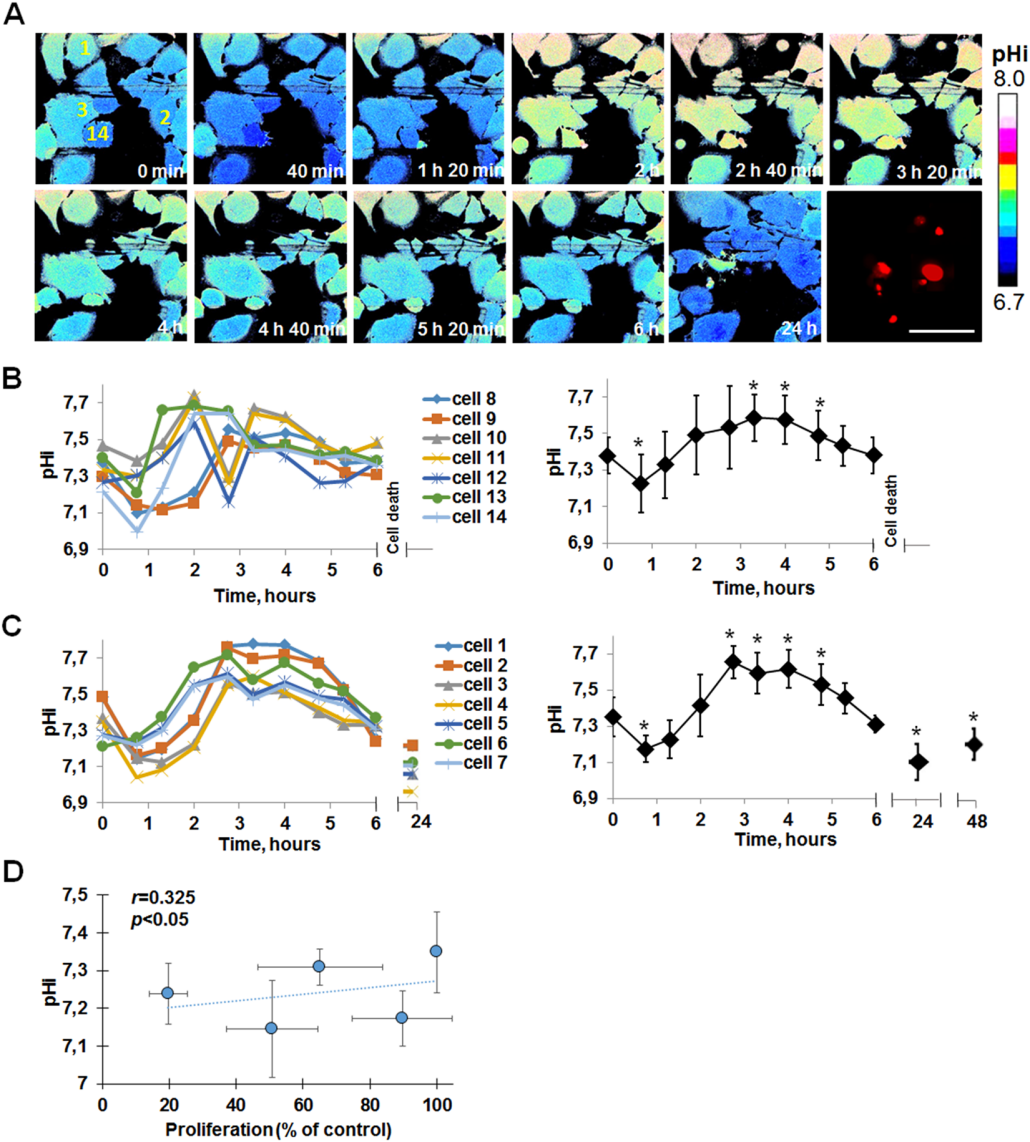
pHi in HeLa-SypHer2 cancer cells under cisplatin exposure. (**A**) Representative time-course pHi imaging during cisplatin exposure and propidium iodide staining at 24 hours. Time after adding cisplatin is indicated on each image. Early changes of pHi in the individual cells and quantification of pHi in cells that further die (**B**) or reduce proliferative activity (**C**). Mean ± SD. In (**B**) n = 75 cells, in (**C**) n = 11 cells. (**D**) Pearson’s correlation between pHi and cell proliferation. Proliferation is expressed as % of untreated control cells counted on the same day. Cell death occurred between 6 and 24 hours of exposure to cisplatin. Monitoring pHi during and at the moment of cell death was out of the scope of this study. The cells indicated by the numbers in (**A**) correspond to the individual cells shown in (**B**,**C**). Bar is 50 µm (applicable to all images). *Statistically significant difference from the initial pHi value, *p* ≤ 0.05.

The initial (i.e. before addition of the drug) pHi was almost identical in both cell subpopulations (7.34 ± 0.10 and 7.38 ± 0.10, respectively). Shortly (45 min) after adding the drug, the pHi decreased in all cells by ~0.2 pH unit (Fig. 2), which indicates an involvement of a non-specific mechanism in early cellular acidification.

Further observation revealed that in the dying cells pHi fluctuated significantly over a period from 80 min to 5 hours (Fig. 2B). Cell death occurred at between 6 and 24 hours of exposure to cisplatin, as confirmed by propidium iodide staining, making further monitoring of the pHi impracticable.

In contrast to the dying cells, a long stable period of alkalization was typical for the cells that further demonstrated a decreased proliferative capacity (Fig. 2C). In these, the pHi value was sharply shifted towards alkaline values by 2–2.5 hours and maintained at an elevated level for up to ~4.5–5 hours, indicating a greater ability of the cells to control pHi homeostasis in the presence of cisplatin. Then the pHi gradually decreased, and at 24 and 48 hours the pHi was more acidic compared to the initial level.

Although pHi oscillations seem to be important in controlling the proliferative capacity of cells^36^, pHi does not correlate with cellular proliferation (*r* = 0.325, Fig. 2D) in the present study that underlies the role of pH homeostatic mechanisms in the cell survival.

Thus, the ability to maintain an alkaline pHi in the presence of cisplatin at short-term exposure was a principal difference between cells that lost proliferation capacity in response to cisplatin and those that died. It is worth mentioning that elicitation of these differences has become feasible owing to monitoring of pHi in the same individual cells. Averaging pHi values for the cells in these subpopulations did not show any difference in pHi dynamics (Fig. 2B,C).

### Metabolic imaging in cancer cells *in vitro* under cisplatin exposure

To access metabolic activity in HeLa cells exposed to cisplatin, the fluorescence intensity-based redox ratio FAD/NAD(P)H and the fluorescence lifetime of NAD(P)H were measured. Separate analysis of metabolic parameters in individual dying and surviving (division-arrested) cells did not reveal any differences between these subpopulations during 6-hour monitoring. Since dead cancer cells lost NAD(P)H and FAD fluorescence, the metabolic measurements were performed only on the viable cells.

**Figure 3:**
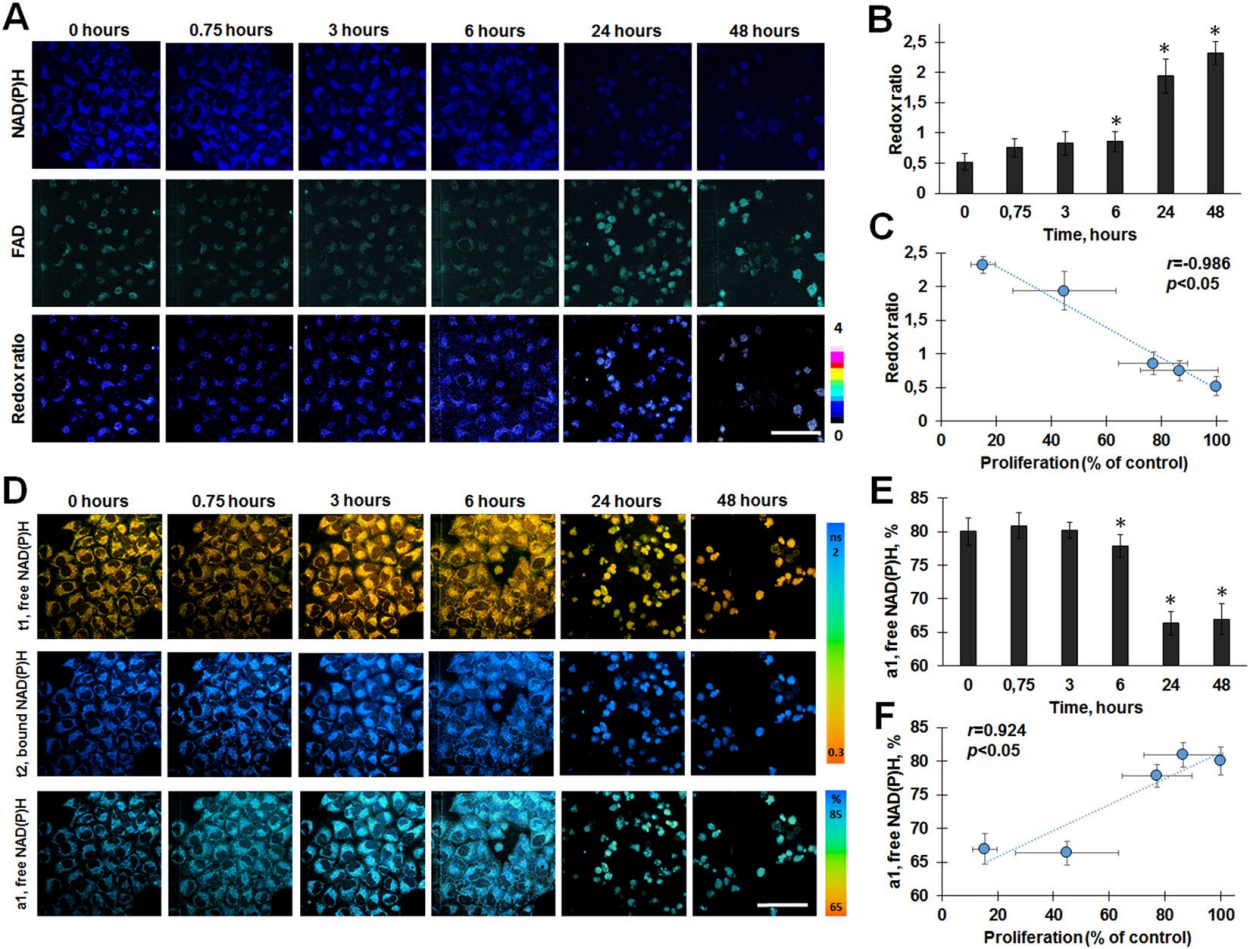
The optical redox ratio FAD/NAD(P)H and FLIM of NAD(P)H in surviving HeLa cells during cisplatin exposure. (**A**) The fluorescence images of NAD(P)H and FAD and the optical redox ratio images. (**B**) Quantification of the redox ratio, mean ± SD, n = 50. (**C**) Pearson’s correlation between the redox ratio and cell proliferation. (**D**) Pseudocolor-coded images of the free (t1, ns) and protein-bound (t2, ns) forms of NAD(P)H and the relative amplitude (a1, %) of free NAD(P)H. (**E**) Dynamics of the fluorescence lifetime amplitude of free NAD(P)H, mean ± SD, n = 50. (**F**) Pearson’s correlation between the relative amplitude (a1, %) of free NAD(P)H and cell proliferation. Bar is 100 µm (applicable to all images). For NAD(P)H: ex. 750 nm, reg. 455–500 nm; for FAD: ex 900 nm, reg. 500–550 nm. *Statistically significant difference from the value before adding cisplatin (0 hours), *p* ≤ 0.05. Dead cells were not included in the analysis because of the loss of their NAD(P)H and FAD fluorescences.

Under exposure to cisplatin we observed a decrease in the fluorescence intensity of NAD(P)H in the HeLa cells and an increase in the fluorescence intensity of FAD, resulting in an increase in the redox ratio (Fig. 3). By 6 hours after adding the drug to the cells a small, statistically significant, increase in the redox ratio was detected (from the 0.52 ± 0.14 of the control to 0.86 ± 0.16, *p* = 0.000) (Fig. 3). A sharp rise of the redox ratio was detected at the 24 hour and 48 hour time-points.

Since, in OXPHOS, NADH is oxidized to NAD+ and FADH is oxidized to FAD+, while in glycolysis and the TCA cycle NAD+ is reduced to NADH, the switch from OXPHOS to a more glycolytic metabolism, typical of rapidly proliferating cancer cells, results in a decrease in the redox ratio^24^. Consequently, the increase in redox ratio observed in our study may indicate decreased cellular metabolic activity in the cells treated with cisplatin.

The spectral characteristics of NADH and its phosphorylated form, NADPH, which plays a central role in the biosynthetic pathways and in antioxidant defense, are indistinguishable; however, bound NADH and bound NADPH can be separated using the fluorescence lifetime imaging (FLIM) technique as their fluorescence lifetimes are markedly different (~1.5 ns for NADH and ~4.4 for NADPH)^37^. Based on the known lifetime differences of NADH and NADPH, we made an attempt to discriminate between their protein-bound forms using the three-exponential fitting model of NAD(P)H fluorescence decay (as described in refs 37 and 38), however, this did not improve the goodness of fit (χ2) across the FLIM images at any time-points of the treatment when compared with the bi-exponential fit. Therefore, we concluded that the concentration of NADPH inside HeLa cells is insufficient for the accurate three-exponential fitting process under our imaging conditions, and so bi-exponential fitting was used to process the FLIM data.

Taking into account that an increased level of NADPH would increase the fluorescence lifetime of protein-bound NAD(P)H^37^, we additionally analyzed the NAD(P)H fluorescence lifetimes and showed that they were the same both before and for over 6 hours after adding cisplatin to HeLa cells (~0.44 ns for the short component, t1, ~2.58 ns for the long component, t2). Further incubation with the drug resulted in a statistically insignificant increase in the fluorescence lifetimes up to 0.48 ns and 2.71 ns, for t1 and t2 respectively (Table 1). The small change in the NAD(P)H fluorescence lifetime by 24 hours of exposure to cisplatin may be caused by a variety of factors, including the lower pH^39^ in the treated cells, changes in the distribution of NADH enzyme binding sites associated with the preferred metabolic pathways^40^, or with a minor increase in NADPH concentration associated, for example, with cisplatin-induced oxidative stress^41^.

**Table 1:** Fluorescence lifetimes of NAD(P)H in HeLa cells before (0 hours) and during the course of 48-hours of treatment with cisplatin. Mean ± SD, n = 75.

	0 hours	0.75 hours	3 hours	6 hours	24 hours	48 hours
t_1,_ ns	0.45 ± 0.01	0.44 ± 0.02	0.44 ± 0.04	0.42 ± 0.02	0.48 ± 0.05	0.48 ± 0.04
t_2,_ ns	2.58 ± 0.11	2.58 ± 0.13	2.58 ± 0.12	2.51 ± 0.24	2.71 ± 0.14	2.72 ± 0.11

The relative amplitude of free NAD(P)H (a1) decreased from 80.02 ± 2.04% to 77.82 ± 1.69% (*p* = 0.00017) after 6 hours, and then down to 66.34 ± 1.71% (*p* = 0.000) by 24 hours (Fig. 3). During the next 24 hours this value did not change further.

As we had confirmed that the contribution of NADPH to the overall fluorescence of NAD(P)H is negligible, we may conclude that the changes in the relative amplitudes of the short and long lifetime components is a result of the reduction in abundance of the protein-free NADH. It is known that free NADH, existing in both the mitochondria and the cytoplasm, is associated with non-oxidative metabolic pathways – glycolysis and the TCA cycle, and a decrease in the fluorescence lifetime amplitude of free NAD(P)H testifies to a shift away from glycolytic toward oxidative metabolism.

We found that the increase in the redox ratio FAD/NAD(P)H and a decrease in the fluorescence lifetime amplitude of free NAD(P)H in HeLa cells treated with cisplatin correlate (*r* = −0.986 and *r* = 0.924, respectively) with decreased cellular proliferation as measured by a viability assay (Fig. 3C,F). Glycolysis is known to promote cancer cell proliferation^8^, it is thereby not surprising that cisplatin-induced decrease in metabolic activity of cells is correlated with their reduced proliferation after the treatment.

### Tumor response to chemotherapy with cisplatin

**Figure 4:**
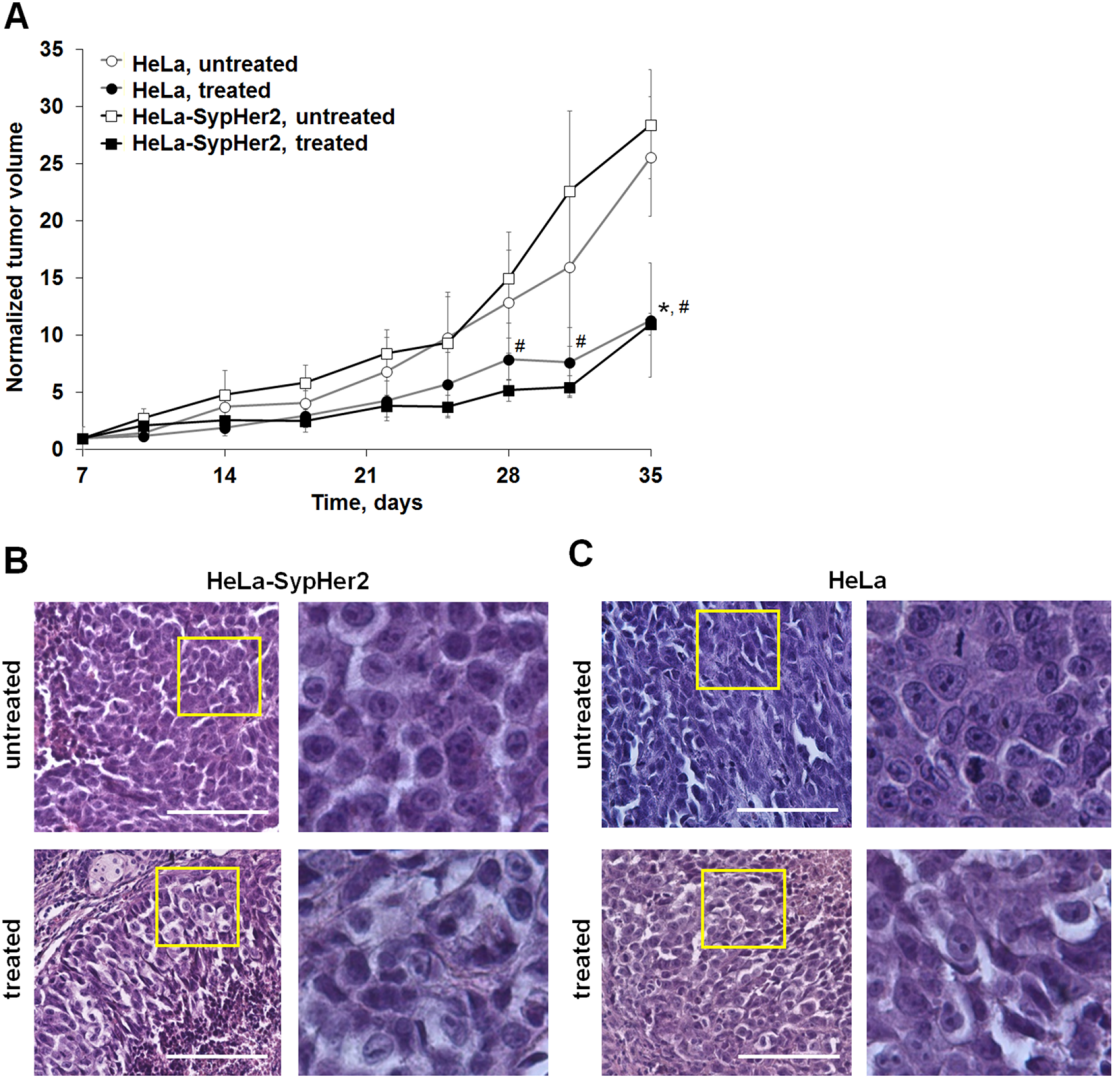
Effects of cisplatin on HeLa tumors in mice. (**A**) Tumor volume dynamics in response to cisplatin. Mice (●, ■) were administrated i.p. with 5 mg/kg cisplatin, totaling 11 doses, for 4 weeks, starting from day 7 after tumor challenge (day 0). Control mice (○, □) received PBS. Mean ± SD, n = 3. Tumor volumes from individual mice were normalized to the values measured on day 7. *Statistically significant difference between treated and untreated HeLa tumors, *p* ≤ 0.05. ^#^Statistically significant difference between treated and untreated HeLa-SypHer2 tumors, *p* ≤ 0.05. (**B**,**C**) Post-treatment histopathology of HeLa-SypHer2 and HeLa tumors. H&E-staining. Enlarged regions are indicated by the yellow squares on the lower-magnification panel (210 × 280 µm, original magnification ×20). Higher-magnification images (60 × 80 µm, original magnification ×40) are shown on the right. Bar is 100 µm.

Monitoring of the HeLa and HeLa-SypHer2 tumor growth during the 35 days after tumor challenge showed that cisplatin treatment (5 mg/kg, totaling 11 doses over 4 weeks) clearly impeded tumor growth. Statistically significant differences between the treated and untreated tumors were observed starting from 28 days in the case of HeLa-SypHer2 and on day 35 in the case of the HeLa tumors (Fig. 4A).

On day 35, 3 days after the final cisplatin dose, the animals were euthanized, and their tumors were analyzed histologically. Untreated tumors had a dense tissue structure and consisted of large polymorphic cells tightly packed together (Fig. 4). Cancer cells had large round or oval nuclei containing fine dispersed chromatin. Lightly basophilic cytoplasm gathered around the nucleus as a thin ring. Mitotic figures were found in 2.29% of cells throughout the tumor nodules. The tumor cells formed complexes surrounded by thin layers of clearly defined connective tissue. Vascularization of the tumors was poor. Typical cancer cells (i.e. without any morphological changes) amounted to ~92% of the total number of cells. Of the rest 3.21% of the cells exhibited dystrophic cellular changes of different degrees of manifestation, and 2.74% of cells had the hallmarks of apoptosis (chromatin condensation, nuclear fragmentation and cytoplasmic eosinophilia).

Three days after the final dose of cisplatin, pronounced dystrophic changes, including voluminous vacuolated cytoplasm, enlarged, clear, swollen nuclei or irregularly shaped small hyperchromatic nuclei and nuclear polymorphism, were found in both the HeLa and HeLa-SypHer2, tumors (Fig. 4). A loss of cell membrane integrity was detected in some cells. Mitoses were infrequent, with the few mitotic cells (0.96% of the total number of cells in the field of view) being identified only at the periphery of the tumor nodes. The number of apoptotic cells had increased to 6.27%. Since most of the cells in cisplatin-treated tumors were enlarged, the number of cells in the field of view had decreased 3-fold compared with untreated tumors. A summary of the histopathologic effects of cisplatin on HeLa tumors is presented in Table 2.

**Table 2:** Histological analysis of tumors treated with cisplatin.

	Unaltered tumor cells, %		Altered tumor cells, %		Total number of cells in the field of view
	Typical cells, %	Mitosis figures, %	Dystrophic changes, %	Apoptosis hallmarks, %	
Untreated	91.76 ± 0.93	2.29 ± 1.18	3.21 ± 0.64	2.74 ± 0.39	169.51 ± 0.71
Treated	28.61 ± 5.52*	0.96 ± 0.09*	64.16 ± 8.59*	6.27 ± 2.98*	52.91 ± 5.51*

*p ≤ 0.05, compared with untreated tumors. Mean ± SD, n = 6 tumors. The percentage of cells with different morphological signs was calculated in 5 randomly selected fields of view for each tumor. The data were *combined from* HeLa and HeLa-SypHer2 tumors.

Therefore, chemotherapy with cisplatin resulted in growth inhibition and multiple cellular changes in HeLa tumor xenografts in mice.

### pHi and metabolic alterations in tumors in response to cisplatin

**Figure 5:**
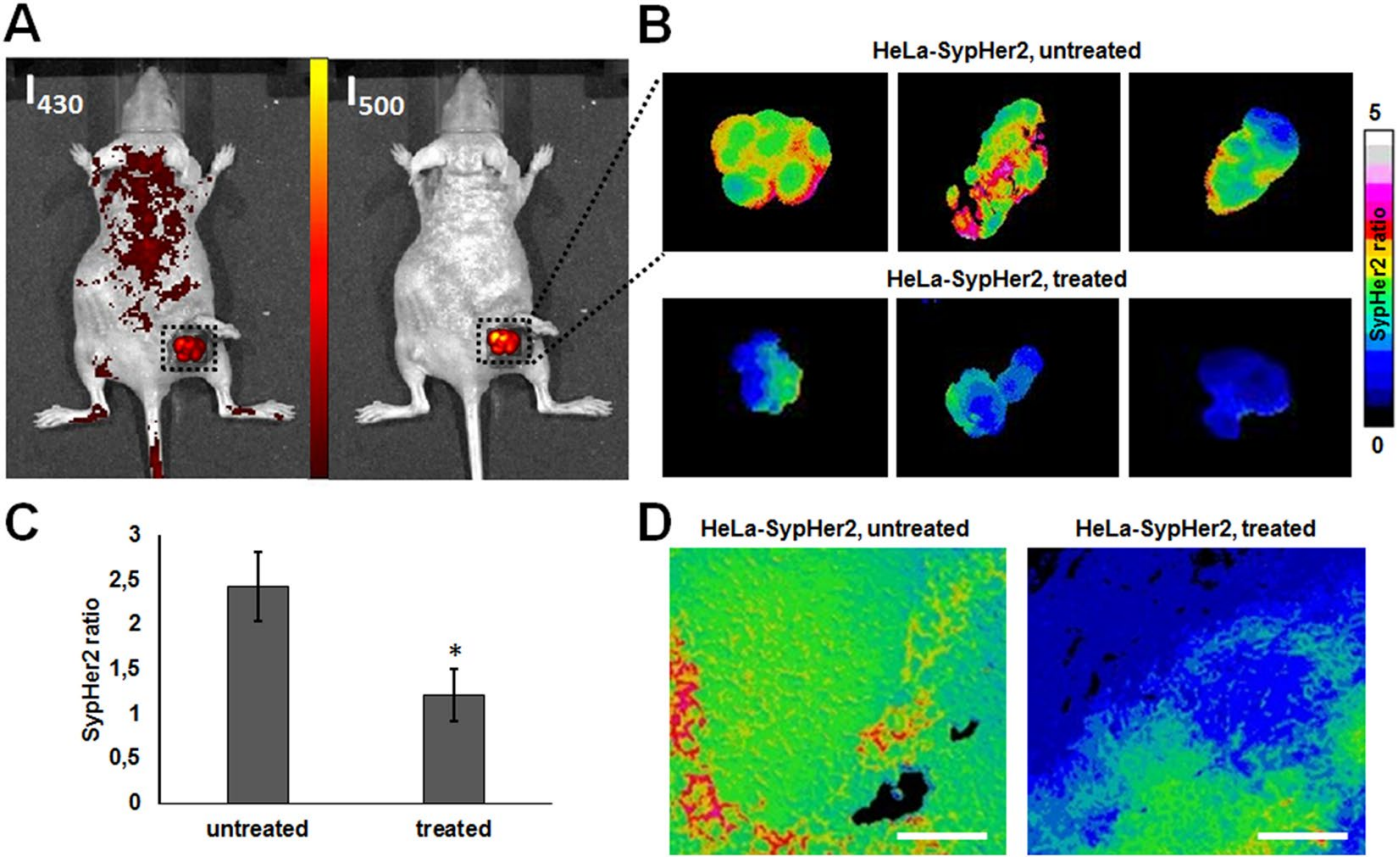
*In vivo* pHi mapping of HeLa-SypHer2 tumors after treatment with cisplatin. (**A**) Fluorescence images with excitation at 430 nm and 500 nm (detection at 540 nm); (**B**) images of SypHer2 ratio (I_500_/I_430_) from three untreated (upper row) and three treated (lower row) tumors *in vivo*; (**C**) quantitative analysis of the SypHer2 ratio in the tumors *in vivo*, mean ± SD, n = 3 tumors; (**D**) SypHer2 ratio from freshly frozen tissue sections. Bar is 100 µm. The cryosections from the HeLa-SypHer2 tumors correspond to the histological slices shown in Fig. 4B. Images were acquired on the 35^th^ day of tumor growth (3 days after the final cisplatin dose). *Statistically significant difference from the untreated control, *p* ≤ 0.05.

pHi was analyzed *in vivo* in HeLa tumors expressing the genetically encoded pH-sensor SypHer2 on day 35 after tumor challenge - 3 days after the final dose of cisplatin (Fig. 5). The SypHer2 fluorescence ratio I_500_/I_430_ was higher in the treated tumors, as compared with the untreated ones (2.43 ± 0.38 *vs* 1.21 ± 0.29, *p* = 0.00001). Although translation of the fluorescence ratio into pH units is impracticable for the tissue *in vivo*, the observed changes in the SypHer2 signal indicate acidification of the pHi (lowering pHi) in the treated tumors.

Fresh cryosections prepared from the SypHer2-expressing tumors displayed a correspondence of the zones with reduced pHi in the treated tumors to the vital, but dystrophically changed tissue. This is consistent with our *in vitro* observations (Fig. 2), where a more acidic pHi was observed in division-arrested cells at long-term exposure to cisplatin.

**Figure 6:**
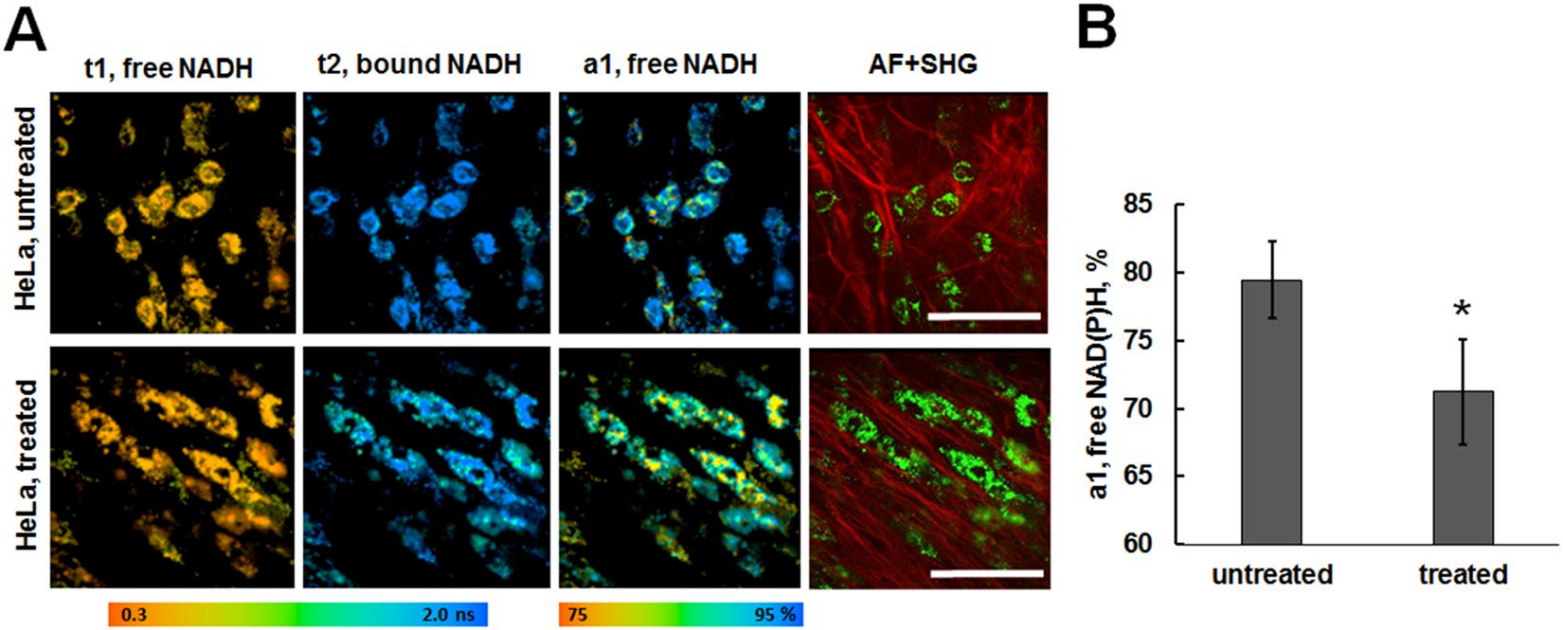
*In vivo* two-photon FLIM of NAD(P)H in HeLa tumors in response to cisplatin. (**A**) Fluorescence lifetimes of NAD(P)H (short t1 and long t2 components), relative amplitude of the short component (free NAD(P)H, a1, %), and overlapping of two-photon excited autofluorescence (AF, green, excitation at 750 nm, detection 410–660 nm) and SHG from collagen (red, excitation at 750 nm, detection - 373–387 nm). (**B**) Relative amplitude a1% of free NAD(P)H calculated from the *in vivo* FLIM images. *Statistically significant difference from the untreated control, *p* ≤ 0.05, mean ± SD, n = 3 tumors (the calculations were made for 50–75 cells in each tumor). FLIM was performed on the 35^th^ day of tumor growth (3 days after the final cisplatin dose). For FLIM of NAD(P)H: ex. 750 nm, reg. 410–660 nm, image collection time was 10 seconds. Bar is 100 µm (applicable to all images).

To identify the metabolic changes induced by cisplatin in HeLa tumors, two-photon FLIM of the metabolic cofactor NAD(P)H was performed *in vivo* after the treatment (Fig. 6). As the fluorescence of FAD was very weak in HeLa tumors, this did not allow an equivalent calculation of its redox ratio. The fluorescence lifetimes of the free (t1) and protein-bound (t2) NAD(P)H measured *in vivo* in untreated tumors were 0.5 ± 0.1 ns and 2.4 ± 0.2 ns, respectively. In the tumors treated with cisplatin, the fluorescence lifetimes did not change and were 0.4 ± 0.1 ns (t1) and 2.3 ± 0.2 ns (t2). It was found that the relative amplitude of free NAD(P)H (a1, %) in cancer cells after chemotherapy decreased in comparison with that in untreated tumors (71.22 ± 3.86% vs 79.48 ± 2.87%, *p* = 0.000). The lower a1 value in the tumors treated with cisplatin probably indicates a shift toward a more oxidative metabolism, similar to that seen in our *in vitro* results.

## Discussion

Current knowledge about the mechanisms of cisplatin action clearly show that its effect is not limited to platinum–DNA adducts, which are formed following uptake of the drug into the nucleus of cells, but several other cellular processes can be activated and mediate the cytotoxicity. Discovering cellular pathways that are affected by cisplatin and/or involved in cellular response may provide us with an important basis for designing new cancer treatment strategies.

In the present study, we explored the relationships between the pHi, cell bioenergetics and the response of cervical cancer to cisplatin. We analyzed in parallel cisplatin-induced pHi and metabolic alterations in living cells and in tumor tissue using advanced fluorescence imaging techniques.

For the first time, we have traced pHi in individual cancer cells in the course of cisplatin treatment, by using a genetically encoded pH-sensor, and this allowed us to identify the differences in dynamics of pHi for the cells with different reactions to cisplatin. A long (~2 hours) period of alkalization of the cellular milieu preceded the cisplatin-induced inhibition of proliferation, while in cells, that subsequently died, the pHi fluctuated. Earlier, an increased pHi has been shown in the cisplatin-resistant cell lines^10–12^. One of the possible mechanisms of more alkaline pHi in the cisplatin-resistant cells as compared with their sensitive counterparts is overexpression of the vacuolar proton pump V-ATPase. Suppression of V-ATPase activity reduces cytosolic pH and potentiates the cytotoxicity of cisplatin *in vitro* ^12, 42^ and *in vivo* ^43^. In general, our results support the hypotheses that the greater ability of cells to control pHi and to maintain alkaline pHi promotes cell survival upon cisplatin treatment.

It is thought that the cytoplasmic pH affects the apoptotic process: pHi alkalinization, a common feature of cancer cells, permits the evasion of apoptosis, whereas pHi acidification is essential for the initiation of apoptosis, particularly, for the activation of caspases and endonucleases and, possibly, for control of the expression of some other actors of the apoptotic cascade^44, 45^. Although, in our research, we identified dead cells without specifying the cell death pathway, cisplatin-induced disturbances of pH homeostasis could potentially determine the cellular response through the modulation of the apoptotic process. A shift toward more alkaline pHi at short-term exposure to cisplatin might favor apoptosis evasion in the cells that further survived but displayed inhibited proliferative capacity.

We found that cisplatin treatment causes a decrease of pHi within the first 1 hour of incubation in all cells, irrespective of the subsequent response of those cells, and at 24 hour- and 48 hour-exposures in viable division-arrested cells. In tumor xenografts treated with cisplatin, pHi was also decreased compared with untreated tumors. The causes of the observed acidification of the cytoplasm are yet to be clarified. It is possible that this effect is associated with cisplatin-induced inhibition of the proton extrusion mechanism(s) due to altered synthesis of the proteins that maintain intracellular pH, and/or the loss their activity. This result is consistent with the study by Rebillard *et al*., where rapid acidification by 0.05 to 0.1 pH unit at 5 and 15 min after cisplatin treatment was demonstrated on HT29 cells^3^. The authors suggest that early intracellular acidification after incubation with cisplatin is due to inhibition of NHE1, an important regulator of pHi, increased activity of which is typical of cancer cells. Although the molecular mechanism of the NHE1 inhibition by cisplatin remain unknown, it seems to be independent of cisplatin-induced DNA-adduct formation. A noncompetitive inhibition of the NHE1 by cisplatin was further confirmed by Milosavljevic *et al*.^46^. To the best of our knowledge, cisplatin-induced lowering pHi has never been shown in tumors *in vivo* before.

It is possible, that the reduction in the pHi in the division-arrested cells favored metabolic reorganization of those cells. A high concentration of cytoplasmic protons is known both to inhibit glycolysis by reducing phosphofructokinase (PFK) activity and the expression of glucose transporters and glycolytic enzymes, while it also activates OXPHOS by promoting the entry of pyruvate and inorganic phosphate into the mitochondrial matrix through ATP synthase^9^.

Using two-photon excitated FLIM of NAD(P)H and FAD, we detected a marked decrease in the fluorescence intensity-based redox-ratio FAD/NAD(P)H and an increase in the relative fluorescence lifetime contribution from protein-bound NAD(P)H in response to cisplatin treatment in viable HeLa cells. Considering NAD(P)H and FAD as biomarkers for metabolic activities of cells^28, 29^, we can speculate that cisplatin-induced changes in their fluorescence can be attributed to the inhibition of glycolysis and/or increased mitochondrial respiration (although alternative metabolic pathways such as the beta oxidation of fatty acids, the pentose phosphate pathway, and glutaminolysis cannot be excluded). A decrease of proliferative capacity of cisplatin-treated cells may partly be the consequence of these metabolic rearrangements.

Previously, inhibition of glycolysis in response to cisplatin has been reported for a variety of cancer cell lines and conditions. For example, in ref. 13 a decreased redox ratio of NADH/FAD and the contribution of free NADH (a1) are shown for head and neck squamous cell carcinoma (HNSCC) *in vitro* in response to cisplatin, which correlates with decreased lactate production/glucose consumption - standard measurements of glycolytic rates, and decreased proliferation. In the *in vivo* study by Shah *et al*., both the redox ratio of NADH/FAD and the fluorescence lifetimes of NAD(P)H of FaDu xenografts decreased 48 hours after cisplatin treatment, and before the tumor decreased in size, indicating that NAD(P)H and FAD fluorescence measurements could reflect early treatment-induced metabolic effects^14^. Bjurberg *et al*. showed *in vivo* an early (on day 1 after intravenous injection of cisplatin) transient increase in FDG glucose analogue uptake in cisplatin-treated SCC tumors in mice, followed by a rapid decrease, confirmed by subsequent tumor regression^15^. In the *in vitro* study by Alborzinia *et al*. the initial metabolic reaction to cisplatin in HT-29, HCT-116, HepG2, and MDA-MB-231 cells was a decrease in mitochondrial respiration immediately after treatment, while at later time-points they observed a decrease in glycolysis^16^. Wang *et al*. showed that cisplatin suppresses breast and cervical cancer cell growth and proliferation by inhibiting cell glucose metabolism^47^. Typically, a decrease in the metabolic (glycolytic) activity of cells in response to cisplatin correlates with reduced cell proliferation and inhibited tumor growth, which is in agreement with our observations.

In principle, a cisplatin-induced shift in tumor metabolism from glycolysis toward OXPHOS is rational, as many enzymes involved in DNA repair, drug efflux, and the maintenance of cellular homeostasis require ATP, and an increase in OXPHOS is, in general, considered a beneficial, adaptive response to DNA damage^48^.

It is worth noticing that, initially, the energy metabolism in HeLa cells is mainly of the oxidative type. As shown in ref. 49, the OXPHOS contribution to the cellular ATP supply predominates over glycolysis, amounting to 77–81% in this cell line. This may partially explain the moderate therapeutic effect of cisplatin on HeLa tumors in mice. Upon continuous treatment for three weeks, none of the treated tumors was completely cured, but all displayed delayed growth. Growth inhibitory effects of cisplatin with similar treatment protocols have been documented in mouse tumor xenograft models of cervical cancer^50–52^. It is likely that the bioenergetics of cells within solid tumors may differ from that in monolayer culture due to the different microenvironment and the nutrient and oxygen availability; however, there is a lack of data about the rates of glycolysis and glucose oxidation in HeLa tumors.

A technical limitation of our study is the impossibility of assessing the oxidative or glycolytic rates using NAD(P)H and FAD fluorescence decay measurements, so additional biochemical assays are required to establish the exact mechanism(s) of the observed alterations in cellular bioenergetics. Nevertheless, the current results indicate that chemotreatment with cisplatin induces metabolic changes in cancer cells *in vitro* and *in vivo*, and that FLIM of metabolic cofactors can be a useful approach for monitoring tumor responses to chemotherapy.

Our present study, therefore, provides more insight into cancer cell response to cisplatin and demonstrates an involvement of pHi and energy metabolism in it. These findings contribute to an understanding of the mechanisms of cisplatin action and of the complex origin of drug resistance.

## Materials and Methods

### Cell cultures

HeLa Kyoto (human cervical carcinoma) cell line, stably expressing the genetically encoded pHi-sensor SypHer2 (HeLa-SypHer2), and its non-expressing counterpart were used. The Hela-SypHer2 cell line was generated and characterized in the Institute of Bioorganic Chemistry of the Russian Academy of Science (Moscow, Russia) and described in our previous paper^16^.

The cells were maintained in DMEM containing 100 µg/ml penicillin, 100 µg/ml streptomycin sulfate and 10% fetal bovine serum (FBS) at 37 °C in a humidified atmosphere with 5% CO_2_.

For registration of the fluorescence signal, cells were plated in glass-bottom GRID-50 dishes (Ibidi, Germany) in complete DMEM medium without phenol red (Life Technologies). The day after plating, the medium was changed to DMEM with 5% FBS, and afterwards the medium was changed every other day.

### Tumor xenografts

All animal protocols were approved by the Ethical Committee of the Nizhny Novgorod State Medical Academy (Russia). All methods were carried out in accordance with relevant guidelines and regulations. Experiments were performed on female athymic nude mice purchased from the Pushchino animal nursery (Pushchino, Russia). Mice of 20–22 g body weight were inoculated subcutaneously in the left flank with HeLa or HeLa-SypHer2 cells (2 × 106 in 200 μL PBS).

Both the FLIM of NAD(P)H and the pHi imaging were implemented *in vivo* on the 35^th^ day of tumor growth. Before the imaging procedures, the mice were anesthetized intramuscularly with a mixture of Zoletil (40 mg/kg, 50 μL, Virbac SA, Carros, France) and 2% Rometar (10 mg/kg, 10 μL, Spofa, Czech Republic) and a skin flap over the tumor was surgically opened. After image acquisition, the animals were sacrificed by cervical dislocation and the tumors were excised for histopathology.

### Chemotherapy

For *in vitro* treatment, cisplatin (Teva) was used in doses of 2.6 µM and 2.4 µM for HeLa and HeLa-SypHer2 cells, respectively, corresponding to inhibitory concentration IC30 as determined by MTT-assay (Fig. S1).

The *in vivo* treatment started when the tumors were ~0.5 cm in diameter (6–7 days after tumor challenge). Six mice were treated with cisplatin (5 mg/kg body weight, in 200 µL PBS, intraperitoneally) three times a week, total 11 doses for 4 weeks. Six untreated animals received 200 µL PBS intraperitoneally on the same days. The tumor size was measured, using a caliper, three times a week, and the volume was calculated as: *V* = *a* * *b* * *b*/2, where a – length of tumor, b – width of tumor.

### Proliferation and viability assays

To assess the proliferative activity, the cells were seeded into 12-well plates at 1 × 10^5^ cells in 1 mL per well. After 24 hours cisplatin was added, and the cells were incubated with the drug for 45 min, 6, 24 or 48 hours. Then the culture medium from each well was collected separately, the cells were harvested with 0.25% trypsin in EDTA, added to the corresponding media, centrifuged at 1000 rpm for 5 min, diluted in 1 mL Hank’s solution, and single-cell suspensions were prepared. To access cell viability, 10 µL of the cell suspension were mixed with 10 µL of trypan blue dye. The total number of cells in the cell suspension and the number of dead cells (trypan blue stained) were calculated using a TC^20^ automated cell counter (Bio-Rad, USA). The data were represented as the number of viable cells and by the percentage of dead cells of the total number of cells.

### Fluorescence microscopy and FLIM

An LSM 710 (Carl Zeiss, Germany) fluorescence confocal laser-scanning microscope equipped with a femtosecond Ti:Sa laser with a repetition rate of 80 MHz and pulse duration of 140 fs, and an FLIM module based on time-correlated single photon counting - Simple Tau 152 TCSPC (Becker & Hickl GmbH, Germany), were used to obtain one- and two-photon fluorescence and FLIM images of the cultured cells. A water immersion objective С-Apochromat 40x/1.2 NA W Korr was used for image acquisition. During image acquisition, the cells were maintained at 37 °C and 5% CO_2_.

For two-photon fluorescence microscopy and FLIM of tumors *in vivo* an MPTflex (JenLab GmbH, Germane) multiphoton tomograph, equipped with a tunable 80 MHz, 200 fs MaiTai Ti:Sa laser and a TCSPC-based FLIM module (Becker & Hickl GmbH, Germany) were used. The images were acquired through a 40x/1.3 NA oil immersion EC Plan-Neofluar objective.

ImageJ 1.39p software (NIH, USA) was used for fluorescence image processing. Analysis of the FLIM data was performed using SPCImage software (Becker & Hickl GmbH, Germany).

### pHi measurement

pHi measurments were performed using the genetically encoded pH-sensor SypHer2. SypHer2 is a ratiometric sensor with dual excitation maxima at 420 nm and 500 nm and an emission maximum at 516 nm^26, 27^. With pH change, the alterations of fluorescence intensity at the two wavelengths are oppositely directed. An acidic pHi is characterized by a low I_500_/I_420_ ratio, while the value of the ratio increases with increasing pHi.

pHi analysis in HeLa-SypHer2 cells *in vitro* was performed before adding cisplatin, then every 40 minutes for the following 6 hours and, finally, after 24 hours. At the 24-hour time-point the cells were stained with iodide propidium according to the manufacture’s protocol, for the verification of dead cells. In a separate study pHi was monitored during the 48 hours after adding the drug. For pHi imaging on the LSM 710 microscope, SypHer2 was excited at a wavelength of 405 nm with a diode laser, and at 488 nm by an argon laser. Emission was detected in the ranges 435–689 nm and 509–689 nm for excitation at 405 nm and 488 nm, respectively. The background signal, taken from an empty region of the image, was subtracted from the measurements, then the ratio of emission intensity resulting from excitation at the two wavelengths was calculated (I_488_/I_405_). To convert the fluorescence ratio to pH units, calibration of the sensor was performed using MOPS buffers and nigericin, as described in ref. 27. All dynamic pHi measurements were carried out in the same cells in 5–7 randomly selected fields of view.

Imaging of pHi in Hela-SypHer2 tumors *in vivo* was performed using an IVIS Spectrum imaging system (Caliper Life Sciences, USA). Fluorescence was excited at two wavelengths: 430 ± 15 nm (I_430_) and 500 ± 15 nm (I_500_), and detected using a 530–550 nm band filter.

After animal sacrifice, the HeLa-SypHer2 tumors were excised and fresh cryosections of the tumor tissue were prepared. For this, each tumor was cut in two parts, one of which was immediately placed into liquid nitrogen and the second into 10% neutral-buffered formalin. Each piece of tumor for cryo-sectioning was then embedded into O.C.T. compound (Optimal Cutting Temperature, Tissue-Tek, Sakura Finetek) on a cryotome holder. Prior to cryosectioning the tumor pieces were brought to −20 °C and allowed to equilibrate for 30 min. The frozen samples were cut into 20 µm thick slices, placed on glass slides and immediately imaged on a Leica DMIL (Leica, Germany) microscope using both CFP ET (Ex: BP 436/20, Em: BP 480/40) and YFP ET (Ex: BP 500/20, Em: BP 535/30) filtration.

### NAD(P)H and FAD registration and analysis

*In vitro* NAD(P)H and FAD two-photon fluorescence was excited at wavelengths of 750 nm or 900 nm and registered in the ranges 455–500 nm or 500–550 nm, respectively. The average power applied to the samples was ~6 mW. The approximate rate of photon counting was 1–2 * 10^5^ photons/second. Image collection time was 60 seconds.

To calculate the optical redox ratio, the fluorescence intensity of FAD was divided by the fluorescence intensity of NAD(P)H. The background signal calculated for a cell-free area of the plate was subtracted. The absence of photobleaching was confirmed by monitoring the photon count rates throughout image acquisition.

The FAD/NAD(P)H redox ratio and the fluorescence lifetime of NAD(P)H were analyzed *in vitro* before adding cisplatin, then at 45 minutes, 3, 6, 24 and 48 hours after addition. Measurements were performed in the same cells in 5–7 randomly selected fields of view.

*In vivo* NAD(P)H fluorescence was excited at a wavelength of 750 nm and detected in the range 410–660 nm using a fixed pre-fitted emission filter. The average power applied to the sample was ~10 mW. Image collection time was 10 seconds. Second harmonic generation was exited at 750 nm and detected in the range 373–387 nm to identify collagen in the tumor tissue.

FLIM measurements were performed only in the cytoplasm of the cells by selecting ~40 × 40 pixel zones as regions of interest. The fluorescence lifetime decay curves of NAD(P)H were fitted to a double-exponential decay model, and the short and long lifetime components (t1 and t2, respectively), and the relative amplitudes of the lifetime components (a1 and a2, where a1 + a2 = 100%) were estimated. The goodness of the fit, the χ2 value, was 0.8–1.2. In a first approximation, for NAD(P)H the first (short, t1) component is attributed to its free, and the second (long, t2) component to its protein-bound state.

### Histopathology

For histological examination, tumors were surgically removed and fixed in 10% neutral-buffered formalin, dehydrated and embedded in paraffin in accordance with standard protocol. 5-μm thick paraffin sections were stained with hematoxylin and eosin (H&E) and examined at 400-fold magnification using a Leica DM2500 microscope (Leica, Japan). The percentages of the unaltered (typical) tumor cells, including mitotic figures, and of altered cells were calculated. The altered cells included any with dystrophic changes (swollen hyperchromic nuclei, vacuolated cytoplasm, and chromatin condensation) and cells with any indication of apoptosis.

### Statistical analysis

The mean values (M) and standard deviations (SD) were calculated to express the data. Student’s t-test and one-way ANOVA with a Bonferroni post-hoc test, where appropriate, were used for data comparison, with *p* ≤ 0.05 being considered statistically significant. The Pearson coefficient (*r*) was used to measure correlations between pHi and proliferation, metabolic parameters and proliferation.
